# Supplementary material for: Dopamine influences attentional rate modulation in Macaque posterior parietal cortex
Source: Sci Rep. 2022 Apr 28;12:6914. doi: 10.1038/s41598-022-10634-w (PMC9050696; doi:10.1038/s41598-022-10634-w)
Supplement: Supplementary file 1 — Supplementary Information. [file 41598_2022_10634_MOESM1_ESM.pdf]

Supplementary Materials

Dopamine influences attentional rate modulation in Macaque posterior parietal cortex

Authors

Jochem van Kempen[1*], Christian Brandt[2], Claudia Distler[3], Mark A. Bellgrove[4], Alexander Thiele[1]

Affiliations

[1] Biosciences Institute, Newcastle University, NE1 7RU, Newcastle upon Tyne, United Kingdom.

[2] Research Unit for ORL – Head & Neck Surgery and Audiology, Odense University Hospital, Odense, Denmark; University of Southern Denmark, Odense, Denmark

[3] Allgemeine Zoologie und Neurobiologie, Ruhr-Universität Bochum, 44801 Germany

[4] Turner Institute for Brain and Mental Health, School of Psychological Sciences, Monash University, Melbourne, Victoria 3800, Australia.

* vankempen.jochem@gmail.com

Dopaminergic drug dose-response curves

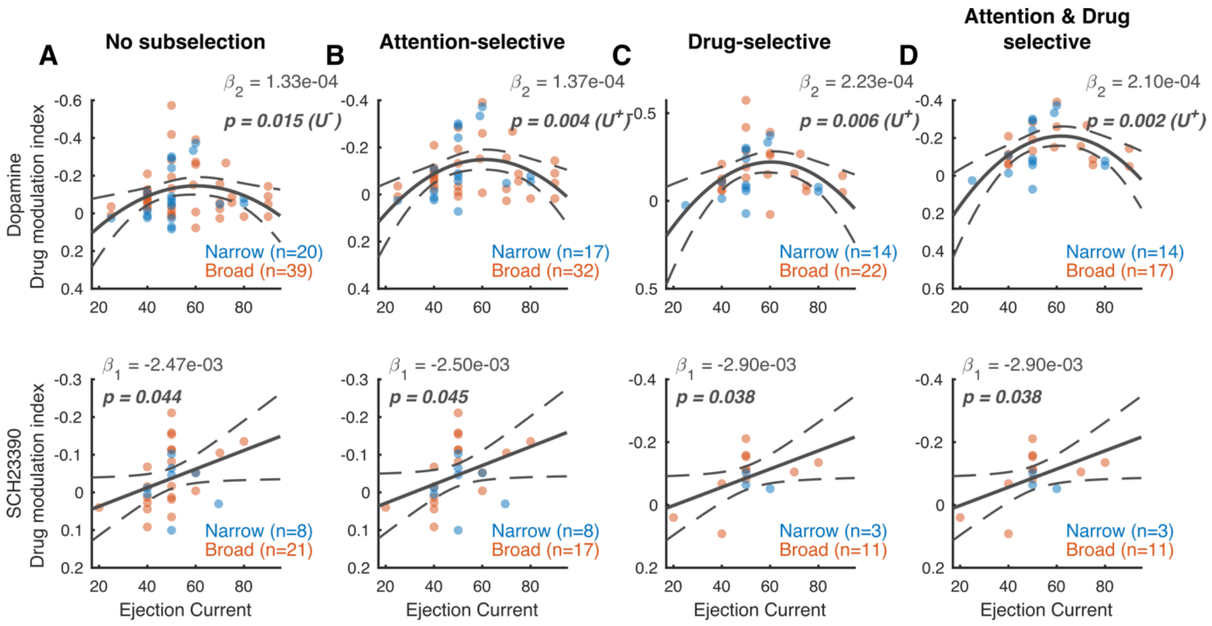

Supplementary Figure S1. Dose-response curve: drug modulation of firing rates. Drug modulation index plotted against ejection current for the non-specific agonist dopamine (top) and the D1R antagonist SCH23390 (bottom) for (**A**) All units (**B**) units that revealed a main or interaction effect for the factor attention (**C**) units that revealed a main or interaction effect for the factor drug and (**D**) units that revealed a main or interaction effect for the factors attention and drug. Note the reversed y-axis. Solid and dotted lines represent significant model fits (applied to all cells simultaneously) and their 95% confidence intervals, respectively. A monotonic relationship is shown if a first-order fit was better than a constant fit, and a non-monotonic relationship is shown if a second-order fit was better than a linear fit. U^+ indicates a significant U-shaped relationship. Statistics: linear mixed-effects model analysis. Statistics deemed significant after multiple comparison correction are displayed in italic and boldface fonts. Note that cells are plotted color coded as 'narrow' and 'broad' spiking, but the analysis of drug-response-curve was based on pooled data.

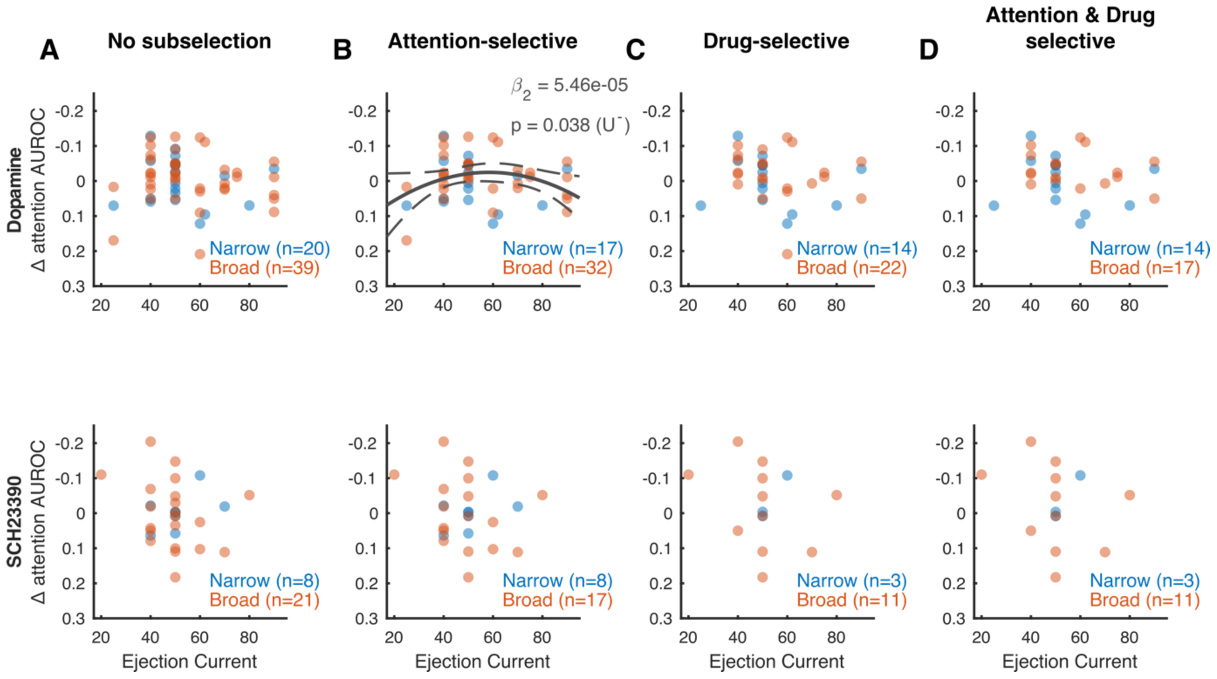

Supplementary Figure S2. Dose-response curve: drug modulation of attention AUROC values. Attention AUROC difference score (drug-no drug) plotted against ejection current for the non-specific agonist dopamine (top) and the D1R antagonist SCH23390 (bottom) for (**A**) All units (**B**) units that revealed a main or interaction effect for the factor attention (**C**) units that revealed a main or interaction effect for the factor drug and (**D**) units that revealed a main or interaction effect for the factors attention and drug. Note the reversed y-axis. Solid and dotted lines represent significant model fits (applied to all cells simultaneously) and their 95% confidence intervals, respectively. A monotonic relationship is shown if a first-order fit was better than a constant fit, and a non-monotonic relationship is shown if a second-order fit was better than a linear fit. U^+ indicates a significant U-shaped relationship. Statistics: linear mixed-effects model analysis. Statistics deemed significant after multiple comparison correction are displayed in italic and boldface fonts. Note that cells are plotted color coded as 'narrow' and 'broad' spiking, but the analysis of drug-response-curve was based on pooled data.

Dopaminergic modulation of different cell types

Dopaminergic drug application modulates firing rates and Fano factors in broad and narrow-spiking units

Cells were classified as narrow or broad-spiking cells according to the median duration of the peak-to-trough time of the spike waveforms (Supplementary Figure S3A & B). These cell types have previously been found to respond differently to dopaminergic drug application in frontal cortex[1,2]. Although narrow and broad-spiking cells have been argued to respectively constitute inhibitory interneurons and excitatory pyramidal cells[3], a more recent study found that output cells in primary motor cortex (unequivocal pyramidal cells) had a narrow action potential waveform[4], and most pyramidal cells in macaque motor cortex express the Kv3.1b potassium channel, associated with the generation of narrow spikes[5]. Therefore, the narrow-broad categorization distinguishes between two different cell type categories, without mapping this classification specifically onto interneurons or pyramidal cells.

We tested whether DA application affected firing rates or rate variability, as quantified by the Fano Factors (FF) and gain variability, measured during the 500 ms preceding the first dimming, using linear mixed-effect models with categorical (effect coded) factors of drug (on/off), attention (RF/away) and unit type (narrow/broad). Confidence intervals were computed across 5000 bootstrap replicates. To control for Type I errors and to aid interpretation of model fit statistics, we additionally report the Kenward-Roger approximation for performing F tests as well as the Bayes factor. We followed these analyses with tests within each unit type, depicted in Supplementary Figure S3 and Supplementary Figure S4. For firing rates, we found a main effect of attention (β = 2.67±0.38, 95% confidence interval = [1.91, 3.45], $\chi^2_{(1)}$ = 29.2, P = 6.44e^{-8}, P_{KR} = 8.19e^{-8}, BF = 6.65e^{6}) reflecting the firing rate increase when attention is directed towards the RF, and a main effect of drug (β = -2.31±0.38, 95% confidence interval = [-3.09 -1.55], $\chi^2_{(1)}$ = 31.1, P = 2.44e^{-8}, P_{KR}

= 3.74e^{-8}, BF = 2.06e^{7}), indicating that DA application reduced firing rates (Supplementary Figure S3C). We did not find a main effect of unit type or any interaction. For FF, we did not find any main effects of attention, drug or unit type, but we found a trending interaction effect between drug and unit type (β = 0.18±0.10, 95% confidence interval = [-0.01 0.38], $\chi^2_{(1)}$ = 2.97, P = 0.084, P_{KR} = 0.09, BF = 1.08) and a three-way interaction between drug, attention and unit type (β = 0.22±0.10, 95% confidence interval = [0.03, 0.42], $\chi^2_{(1)}$ = 4.75, P = 0.029, P_{KR} = 0.036, BF = 3.37). This interaction reflects that when attention is directed towards the RF, DA application increases FF, whereas when attention is directed away from the RF, DA application decreases FF in narrow-spiking units (Supplementary Figure S3D).

We performed the same analyses for the application of SCH23390. For firing rates, we found a main effect of attention (β = 3.33±0.50, 95% confidence interval = [2.33, 4.30], $\chi^2_{(1)}$ = 20.9, P = 4.92e^{-6}, P_{KR} = 7.21e^{-6}, BF = 3.22e^{4}) reflecting the firing rate increase when attention is directed towards the RF, and a main effect of drug (β = -1.29±0.50, 95% confidence interval = [-2.3, -0.29], $\chi^2_{(1)}$ = 8.47, P = 0.004, P_{KR} = 0.005, BF = 13.3), indicating that SCH23390 application reduced firing rates (Supplementary Figure S3E). We additionally found an interaction between attention and unit type (β = 1.35±0.50, 95% confidence interval = [0.37, 2.33], $\chi^2_{(1)}$ = 6.72, P = 0.01, P_{KR} = 0.014, BF = 4.9), indicating that narrow-spiking units increased their firing rates more when attention was directed towards the RF. We did not find any effect of drug application or attention for FF, but we found a trending main effect of unit type (β = 0.85±0.42, 95% confidence interval = [-0.002, 1.69], $\chi^2_{(1)}$ = 3.49, P = 0.06, P_{KR} = 0.09, BF = 0.19). However, the lack of clear significant effects in conjunction with the low number of narrow-spiking units for this sample raise doubts about their robustness (Supplementary Figure S3F).

Dopaminergic drug application modulates gain variability

We next investigated the effects of drug application and attention on gain variability[6]. Neural activity often displays super-Poisson variability (larger variance than the mean), resulting from trial-to-trial changes in excitability, that can be modeled by fitting a negative binomial distribution to the spike rate histogram. This distribution is characterized by a dispersion parameter that captures this additional variability and has been proposed to reflect stimulus-independent modulatory influences on excitability[6]. Whereas FF is a measure of variability that is accurate when the variance is proportional to the mean, gain variability captures the nonlinear variance-to-mean relationship[7]. During DA application we found a trending main effect of attention (β = -0.1±0.041, 95% confidence interval = [-0.18, -0.02], $\chi^2_{(1)}$ = 3.26, P = 0.07, P_{KR} = 0.07, BF = 0.6) and a main effect of drug application (β = 0.20±0.041, 95% confidence interval = [0.12, 0.28], $\chi^2_{(1)}$ = 18.5, P = 1.72e^{-5}, P_{KR} = 2.33e^{-5}, BF = 1.38e^{4}) on gain variability. This indicates increased variability during drug application and decreased variability when attention was directed towards the RF. We furthermore found a trending interaction between attention and unit type (β = -0.07±0.041, 95% confidence interval = [-0.15, 0.01], $\chi^2_{(1)}$ = 2.72, P = 0.099, P_{KR} = 0.11, BF = 0.65), revealing a decrease in gain variability in narrow-spiking units when attention was directed towards the RF (Supplementary Figure S4A). For SCH23390, we found a trending main effect of attention (β = -0.14±0.081, 95% confidence interval = [-0.3, 0.02], $\chi^2_{(1)}$ = 3.52, P = 0.061, P_{KR} = 0.065, BF = 1.08) and a main effect of drug application (β = 0.16±0.081, 95% confidence interval = [0.0004, 0.32], $\chi^2_{(1)}$ = 9.04, P = 0.003, P_{KR} = 0.004, BF = 37), indicating increased gain variability with drug application and decreased variability when attention was directed towards the RF. In addition, there was as a trending interaction effect between drug application and unit type (β = 0.16±0.081, 95% confidence interval = [-0.31, 0.001], $\chi^2_{(1)}$ = 3.56, P = 0.059, P_{KR} = 0.08, BF = 1.33), indicating a relatively larger difference in gain

variability in broad compared to narrow-spiking units. The model fits within each unit type revealed a significant main effect of drug application (β = 0.31±0.088, p = 0.0009) and an interaction between drug application and attention (β = 0.18±0.088, p = 0.048) for broad-spiking units. For narrow-spiking units we found a main effect of attention (β = -0.14±0.03, p = 0.001) and a trending interaction effect between drug application and attention (β = 0.06 ±0.03, p = 0.071) (Supplementary Figure S4B).

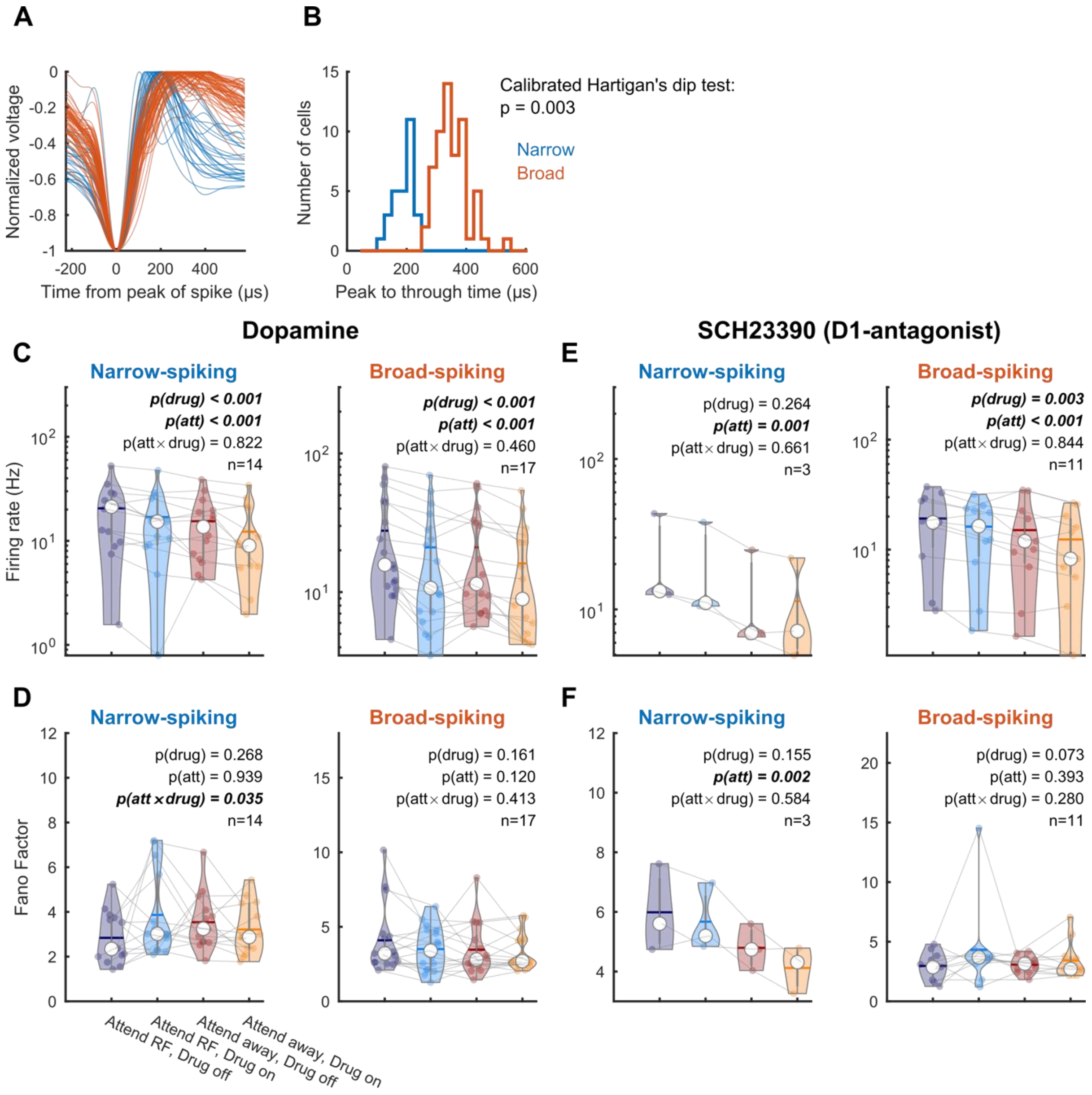

Supplementary Figure S3. Dopaminergic modulation of firing rates across broad and narrow-spiking units. (**A**) Average spike waveforms for the population of units. (**B**) Distribution of peak-to-trough ratios. Statistics: calibrated Hartigan's dip test [8]. (**C**) Average firing rates between attention and drug conditions for the non-specific agonist dopamine for narrow-spiking (left) and broad-spiking (right) units. (**D**) Fano factors between attention and drug conditions for the non-specific agonist dopamine. (**E-F)** Same conventions as (**C-D**) but for the D1R antagonist SCH23390. Only units that revealed a main or interaction effect for the factors drug and attention were included in this analysis. Individual markers represent the average firing rate or Fano Factor for a single unit. The white marker denotes the median and the error bars the interquartile range. Horizontal bars denote the mean. Statistics: linear mixed-effect models.

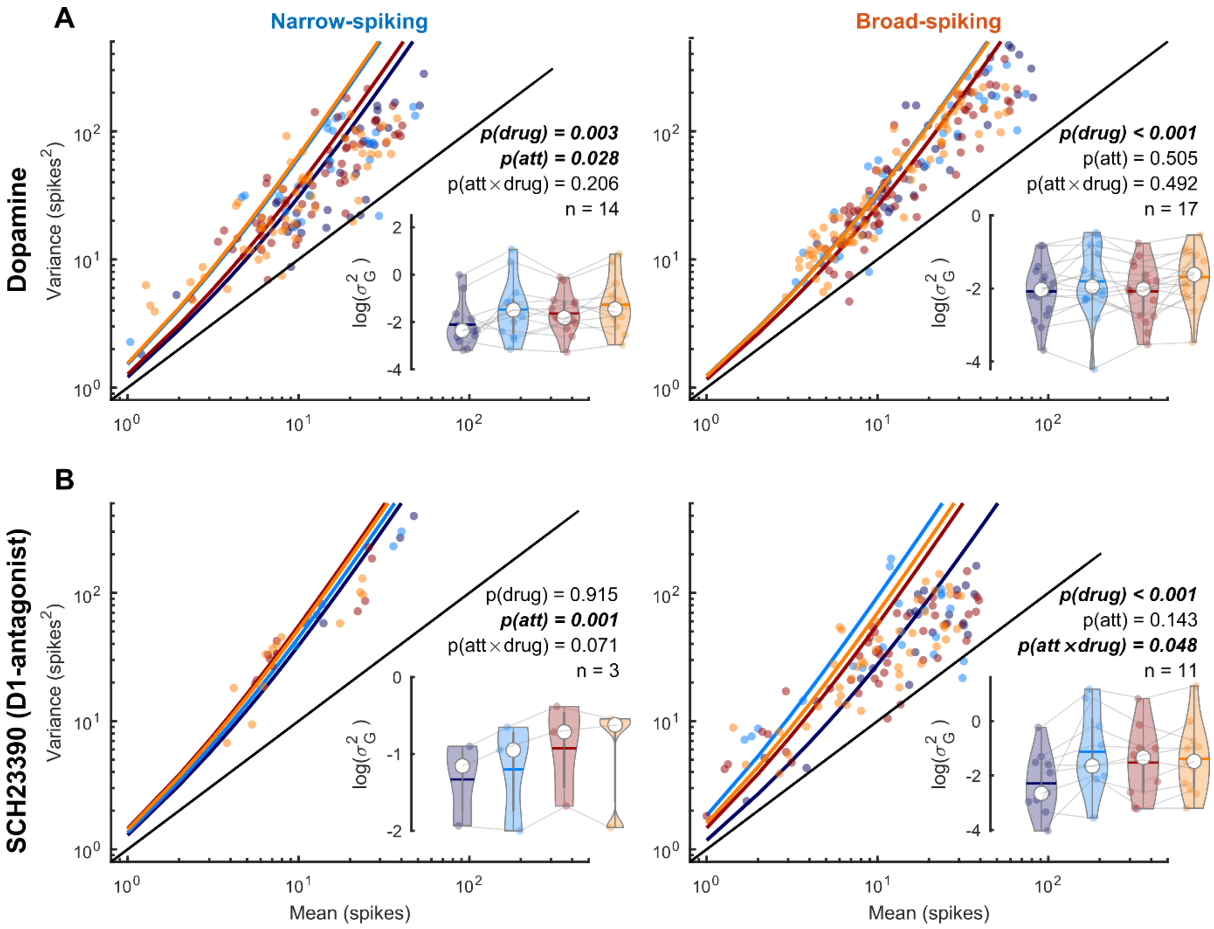

Supplementary Figure S4. Dopaminergic modulation of gain variability across broad and narrow-spiking units. (**A**) Variance-to-mean relationship across attention and drug conditions for narrow-spiking (left) and broad-spiking (right) units for the non-specific agonist dopamine. Individual dots depict the variance and mean across

trials for a single condition. Solid lines show the predicted mean-to-variance relationship given the average fitted dispersion parameter (σ_G^2). Insets show σ_G^2 for each unit and their comparison across attention and drug conditions. Individual markers represent the gain variability for a single unit. The white marker denotes the median and the error bars the interquartile range. Horizontal bars denote the mean. (**B**) Same conventions as (**A**) but for the D1R antagonist SCH23390. Only units that revealed a main or interaction effect for the factors drug were included in this analysis. Statistics: linear mixed-effect models.

Dopaminergic drug application modulates attention AUROC in broad-spiking units

To investigate whether DA affected attention-specific activity, we tested if attention AUROC values were modulated by drug application. Drug application reduced AUROC values for broad-spiking cells, whereas narrow-spiking cells were unaffected (Supplementary Figure S5A) [two-sided Wilcoxon signed-rank test; narrow-spiking: Δ-AUROC -0.002±0.01, p=0.952, Cohen's d=0.030; broad-spiking: Δ-AUROC -0.034±0.006, p=0.009, Cohen's d=-0.70]. Corrected AUROC values (1-AUROC if the AUROC value was smaller than 0.5 without drug application, Supplementary Figure S5) revealed a trending relationship [two-sided Wilcoxon signed-rank test; broad-spiking: Δ-AUROC -0.02±0.01, p=0.08, Cohen's d=-0.38]. SCH23390 application did not modulate AUROC values for either cell type (Supplementary Figure S5B). DA thus had a cell-type specific effect on attentional rate modulation, but this was only trending, once corrected values of AUROCs were used.

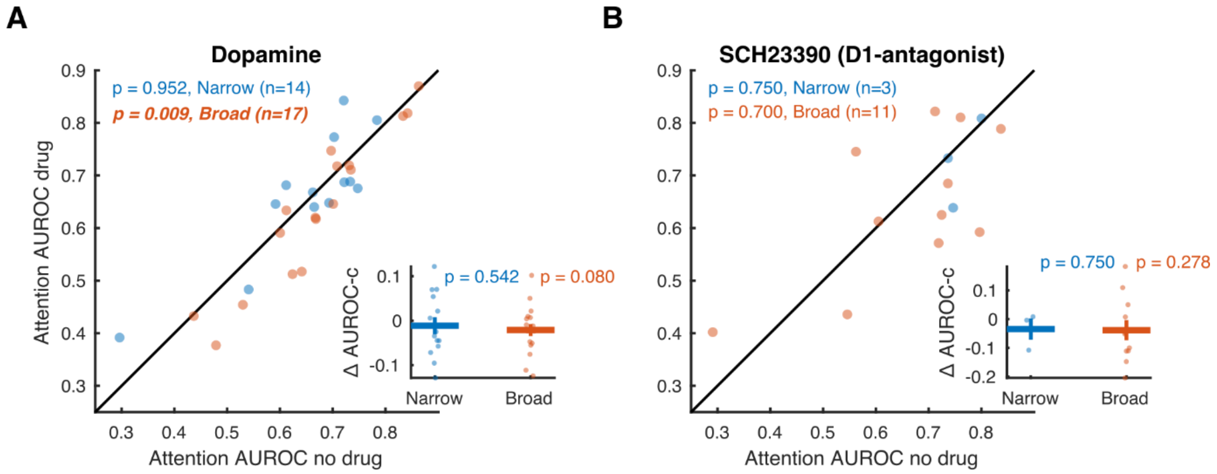

Supplementary Figure S5. Dopaminergic modulation of AUROC values for broad and narrow-spiking units (**A**-**B**) Area under the receiver operating characteristic (AUROC) curve between no drug and drug conditions for the non-specific agonist dopamine (**A**) and the D1R antagonist SCH23390 (**B**). The insets depict the difference (drug-no drug) of the corrected AUROC values (Materials & Methods). Only cells that revealed a main or interaction effect for the factors of drug and attention were included in this analysis. Statistics: Wilcoxon signed rank tests (FDR corrected). Statistics deemed significant after multiple comparison correction are displayed in italic and boldface fonts.

As a note of caution, our subdivision into broad and narrow spiking cells included some multi-unit recordings, whereby we (subjectively) estimate that these contained spikes from maybe 2-4 cells (estimate is based on quality of isolation in conjunction with firing rates [the more cells the higher the firing rate]). How could such multi-unit inclusion affect our results? We argue that it depends on how 'multi' these multi-units are. If (*example A*) multi-units consist of 2 cells, one narrow spiking, the other broad spiking, (and assuming the narrow spiking is also fast spiking), then this multi-unit would most likely be classified as narrow spiking as more spikes from the fast-spiking neuron contribute to classification (and hence the median spike width would be of narrow type). However, if (*example B*) there were 4-5 neurons contributing to the multi-unit, then on average ~3-4 would be broad spiking (assuming 20% of neurons are narrow spiking and assuming unbiased sampling at the electrode [which is not the case, bias is towards oversampling larger soma cells]), while ~1

contributing cell would be of narrow spiking type. In this case the multi-unit would most likely be classified as broad spiking, as most action potentials would stem from broad spiking cells.

In our sample, broad spiking cells are the ones where DA affects attentional modulation in the form of AUROC, Fano-Factor, or gain variability (while narrow spiking ones are not). In the case a multi-unit was of *example A*, then a broad spiking cell (where attentional modulations are affected by DA) would contribute some effects to the overall sample of narrow spiking cells (but not enough to make it significant). However, in *example B* a narrow spiking cell (with does not show DA effects) would reduce the effect that is otherwise present in broad spiking cells. Given this reasoning, the effects reported in supplementary materials might be a conservative estimate of the effects that are really present. However, we admit that this is rather speculative. We also note that the sample sizes after splitting cells into different types is rather small and hence the results are to be treated with caution. We nevertheless feel they are worthy of reporting, as a possible reference for future studies.

Supplementary references

1. Jacob, S. N., Ott, T. & Nieder, A. Dopamine regulates two classes of primate prefrontal neurons that represent sensory signals. *J. Neurosci.* **33**, 13724–34 (2013).

2. Jacob, S. N., Stalter, M. & Nieder, A. Cell-type-specific modulation of targets and distractors by dopamine D1 receptors in primate prefrontal cortex. *Nat. Commun.* (2016) doi:10.1038/ncomms13218.

3. Mitchell, J. F., Sundberg, K. a & Reynolds, J. H. Differential Attention-Dependent Response Modulation across Cell Classes in Macaque Visual Area V4. *Neuron* **55**, 131–141 (2007).

4. Vigneswaran, G., Kraskov, A. & Lemon, R. N. Large Identified Pyramidal Cells in Macaque Motor and Premotor Cortex Exhibit 'Thin Spikes': Implications for Cell Type Classification. *J. Neurosci.* **31**, 14235–14242 (2011).

5. Soares, D. *et al.* Expression of Kv3.1b potassium channel is widespread in macaque motor cortex pyramidal cells: A histological comparison between rat and macaque. *J. Comp. Neurol.* **525**, 2164–2174 (2017).

6. Goris, R. L. T., Movshon, J. A. & Simoncelli, E. P. Partitioning neuronal variability. *Nat. Neurosci.* **17**, 858–865 (2014).

7. Thiele, A. *et al.* Attention Induced Gain Stabilization in Broad and Narrow-Spiking Cells in the Frontal Eye-Field of Macaque Monkeys. *J. Neurosci.* **36**, 7601–12 (2016).

8. Ardid, S. *et al.* Mapping of functionally characterized cell classes onto canonical circuit operations in primate prefrontal cortex. *J. Neurosci.* (2015) doi:10.1523/JNEUROSCI.2700-14.2015.
